# *CT-Finder*: A Web Service for CRISPR Optimal Target Prediction and Visualization

**DOI:** 10.1038/srep25516

**Published:** 2016-05-23

**Authors:** Houxiang Zhu, Lauren Misel, Mitchell Graham, Michael L. Robinson, Chun Liang

**Affiliations:** 1 Department of Biology, Miami University, Oxford, Ohio, 45056, USA; 2 Department of Software Engineering, Miami University, Oxford, Ohio 45056, USA

## Abstract

The CRISPR system holds much promise for successful genome engineering, but therapeutic, industrial, and research applications will place high demand on improving the specificity and efficiency of this tool. *CT-Finder* (http://bioinfolab.miamioh.edu/ct-finder) is a web service to help users design guide RNAs (gRNAs) optimized for specificity. *CT-Finder* accommodates the original single-gRNA Cas9 system and two specificity-enhancing paired-gRNA systems: Cas9 D10A nickases (Cas9n) and dimeric RNA-guided FokI nucleases (RFNs). Optimal target candidates can be chosen based on the minimization of predicted off-target effects. Graphical visualization of on-target and off-target sites in the genome is provided for target validation. Major model organisms are covered by this web service.

Cas nucleases, derived from bacterial adaptive immune systems, possess the ability to cause double-strand breaks at targeted locations in genomes. These double-strand breaks in the genome may be repaired either through non-homologous end joining, causing insertions or deletions (indels) that are expected to result in gene knockout, or through homology-directed repair, in which a repair template is provided for insertion at the location of the break^1^. Unlike transcription activator-like effector nucleases (TALENs) and zinc finger nucleases (ZFNs) – tools for similarly sophisticated genomic editing – the CRISPR/Cas system is highly adaptable and easily programmed. This is because CRISPR/Cas eliminates the need for protein engineering, and in turn only requires the design of a simple and short (~20 *nt*) guide RNA (gRNA) that base pairs with the intended on-target genomic site^2^. Another advantage of CRISPR/Cas is its ability to create multiple simultaneous mutations, as demonstrated in mouse embryonic stem cells^3^.

Unfortunately, the original Cas9 system raises issues of specificity due to the shortness of the gRNA sequence. Even with a gRNA sequence that is entirely unique within the genome of interest, Cas nucleases may still cleave at unintended off-target sites that bear very high similarity to the target DNA sequence. Off-target site sequences resemble the target site sequences except for the presence of indels, mismatches, or a combination. Off-target cleavage is especially concerning to potential future therapeutic applications of genome editing; it is crucial to reduce the possibility of off-target cleavage to the point of virtual elimination before performing genomic editing on human subjects.

As an improvement over the Cas9 system, the Cas9 D10A nickases (Cas9n) system operates on the biological principle that two single-strand breaks in close proximity are equivocal to a double-strand break. Two nickases are equipped with non-identical gRNAs designed to match to two proximal DNA targets. The combination of two single-strand, approximately simultaneous breaks acts to achieve cleavage activity at the target site^4^. This strategy has been demonstrated to reduce off-target effects by a factor of 50- to 1,500-fold as compared to Cas9^4^. Although single-strand breaks at off-target sites are normally repaired with high fidelity via the base excision repair (BER) pathway^5^, concerns have arisen that these nickases could individually introduce indel mutations at off-target sites with high efficiency^6^.

Meanwhile, RNA-guided FokI nucleases (RFNs) extend the concept of paired gRNA systems to a level of a true dimeric system. RFNs, like the Cas9n system, use two gRNAs. Unlike Cas9n, which can cause single-strand breaks independently, RFNs strictly require that both gRNAs must be present as a pair with an appropriate distance of space between them. The spacer length between two gRNAs in the RFNs system is 14–17 *nt*^7^. The stringency of the requirements for RFNs results in acutely specific targeting, essentially eliminating Cas9-induced off-target mutagenesis, but comes at the disadvantage of reduced target space and thus narrower applicability. Both Cas9n and RFNs systems utilize paired gRNAs and claim high specificity of gene editing due to the effective doubling of the number of base pairs matched to the target sites, but have a more limited set of possible target candidates as compared to Cas9.

So far, there are a few web services, including CRISPR DESIGN^8^, E-CRISP^9^, GT-Scan^10^ and CRISPRdirect^11^, available for helping biologists design gRNAs and determine target sites for CRISPR systems. To the best of our knowledge, there is no web service designed to accommodate three different types (Cas9, Cas9n and RFNs) of genome editing CRISPR systems. We have designed and developed CRISPR Target Finder (*CT-Finder*) to cover Cas9, Cas9n and RFNs systems and aid researchers in maximizing target specificity in the application of CRISPR technologies. For each target candidate in any of the three CRISPR systems, *CT-Finder* is able to predict off-target sites in the genomic region. This allows for the optimal target candidates to be chosen based on the minimization of predicted off-target effects. Differently from other online tools for CRISPR systems, *CT-Finder* incorporates a high degree of flexibility while maintaining a simple graphical interface that is able to accommodate user inputs for many of the most important features, such as the gRNA length and protospacer adjacent motif (PAM) sequence. Compared with other tools, *CT-Finder* is more comprehensive and precise in its determination of possible off-target sites, as it considers mismatches, insertions, and deletions separately for both the seed and non-seed regions. Additionally, *CT-Finder* is able to provide users with more information (*e.g.*, GC content of the entire target sequence excluding the PAM sequence, and GC content of the 6 nucleotides closest to the PAM sequence, which has been found to be a significant predictor of gRNA efficiency). *CT-Finder* also introduces, for the first time, a smooth, scrollable genome browser for visualizing on-target and off-target sites in the genomic context, allowing users to validate data and consider gene annotation features when choosing proper targets.

## Design Rationale

The Cas nucleases of CRISPR systems consist of an RNA binding domain, a α-helical recognition lobe, a nuclease lobe, and a PAM-interacting site^12^. The nuclease lobe in Cas9 contains two DNA-cleaving nuclease domains: RuvC and HNH, each of which cleaves one strand of the targeted DNA^12^. Together, these two domains cause a double-strand break. Cas9n is modified with a mutation in one of these domains, which in turn causes only single-strand breaks^4^. The PAM, recognized by the PAM-interacting site of Cas, is critical to binding of Cas to the target site. The PAM sequence is not a part of the gRNA, but must be present immediately at the 3′ end of the targeted DNA site^12^. Mismatches in the PAM sequence are so poorly tolerated that they are typically expected to result in elimination of detectable target activity by Cas; in fact, base pair mutations in the PAM sequence are a viral defense against microbial CRISPR systems^13^. In *Streptococcus pyogenes* Cas9 (SpCas9) system, the default on-target PAM sequence is *NGG* and the default off-target PAM sequence is *NRG*, including *NGG* and *NAG*. The off-target PAM sequence *NAG* is not adequately efficient to be considered for on-target, but presents appreciable efficacy to be considered for off-target sites. Therefore, flexibility in choice of on-target and off-target PAM sequences is incorporated into *CT-Finder*.

*CT-Finder* is designed to be unrestricted by the gRNA length. Though many current online tools for choosing gRNAs rely on an inflexible 20-*nt* standard gRNA length, it is known that there is a range of possible effective gRNA lengths for CRISPR/Cas. The natural length of SpCas9 gRNA is 20 *nt*, but the range of permissibly effective lengths varies between 17 and 20 *nt*. Length of gRNAs also varies across different Cas9 systems; for example, *Staphylococcus aureus* Cas9 (SaCas9) in mammalian cells is reported to exhibit the greatest editing efficiency with a gRNA length of 21 to 23 *nt*^14^. Length does appear to be a determinant of cleavage efficiency and potentially also off-target cleavage risk. Truncated gRNAs 17 or 19 *nt* in length, for example, have been demonstrated in SpCas9 to have significantly reduced off-target mutagenesis in mammalian cells while retaining cleavage efficiency comparable to canonical 20-*nt* gRNA^15^. Therefore, the flexibility in gRNA length offered by *CT-Finder* enables more applications in genome editing and addresses potential species-specific needs.

Total number of mismatched base pairs is a key factor for cleavage efficiency of SpCas9 CRISPR systems^8^. As a general rule, mismatches at the 3′ end of the gRNA, proximal to the PAM sequence, are less tolerated than mismatches closer to the 5′ end of the gRNA^8^. In SpCas9, 2 concatenated or interspersed mismatches considerably reduce cleavage efficiency; 3 concatenated mismatches cause reduction beyond that of 2 mismatches; and 3 or more interspersed mismatches or 5 concatenated mismatches result in elimination of detectable SpCas9 activity in the vast majority of tested genomic loci^8^. Insertions and deletions are somewhat more complex. Cas9 tolerates single gRNA bulges up to 4 *nt* in length^16^. Cas9n has been found to tolerate both single gRNA and DNA bulges in one of the guide strands, when one bulge-forming gRNA is utilized with a perfectly matched gRNA^16^. Accordingly, *CT-Finder* takes into account insertions, deletions, and mismatches separately in the seed and non-seed regions, and also allows users to set the maximum number of strands (*i.e.*, 0, 1, or 2) that can tolerate bulges in Cas9n and RFNs systems.

Even when comparing single gRNAs targeting the same gene, substantial variance in efficacy exists, indicating the importance of the choice of sequences^17^. *CT-Finder* includes GC-content calculations as a measure of target candidate efficiency. In mammalian cell lines, medium GC content in 20-*nt* single gRNAs is more efficient than low or high GC-content single gRNAs^17^,^18^. *CT-Finder* also includes a measure of GC content of the 6 nucleotides proximal to the PAM sequence in each target candidate, as a study in *Drosophila* found a strong positive correlation between efficiency and single gRNA GC content of the 6 nucleotides proximal to the PAM sequence^18^. Experimental evidence indicates that it is critical for these 6 nucleotides to possess a GC content of >50%, *i.e.* 4 or more of them are either G or C - but no further increases in efficiency are seen past this threshold^18^. This has such a significant effect on efficiency that simultaneous mutation of multiple genes is achievable with single gRNAs of high GC content in the 6 nucleotides adjacent to the PAM sequence; 4 genes have been mutated in one step experimentally in a fly model^18^. In *CT-Finder*, GC contents of both the 6 nucleotides proximal to the PAM and the entire gRNA are provided for users to consider when choosing the most efficient target sites.

Among the existing web services for CRISPR systems, none seamlessly integrate data visualization in terms of genomic positions of on-target and off-target sites along with associated gene structures within a genome browser. In *CT-Finder*, we integrated JBrowse^19^, a JavaScript-based genome browser, for visualization of predicted on-target and off-target sites. JBrowse enables customizability to the visualization by permitting users to build upon the reference sequence annotation through the addition of multiple genome feature tracks^19^. Compared to its predecessor GBrowse, JBrowse is exceptionally fast and provides smooth, continuous movement along the genome. This seamless data integration and visualization using JBrowse is important for biologists to validate data and to identify optimal target sites.

## Graphic User Interface

**Figure 1:**
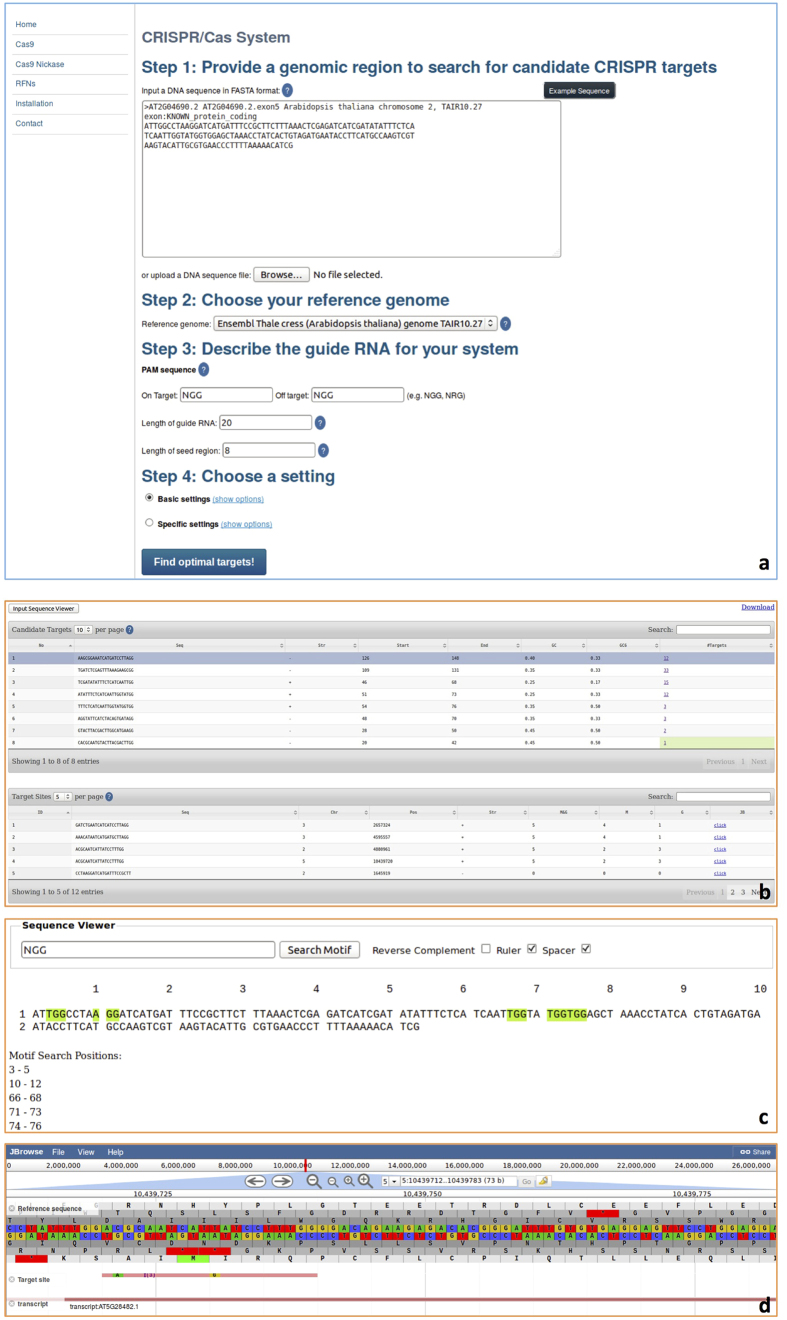
Example settings and result pages for the Cas9 system. Panel (**a)** the settings page for the Cas9 system. Panel (**b)** Table Viewer, which displays a list of target candidates (top) and a list of corresponding target sites in the genome for each target candidate (bottom). Panel (**c)** Sequence Viewer, which shows the user input sequence and highlights the subsequence or pattern specified by a user. Panel (**d)** Genome Browser, which visually displays the target sites and gene annotation.

**Figure 2:**
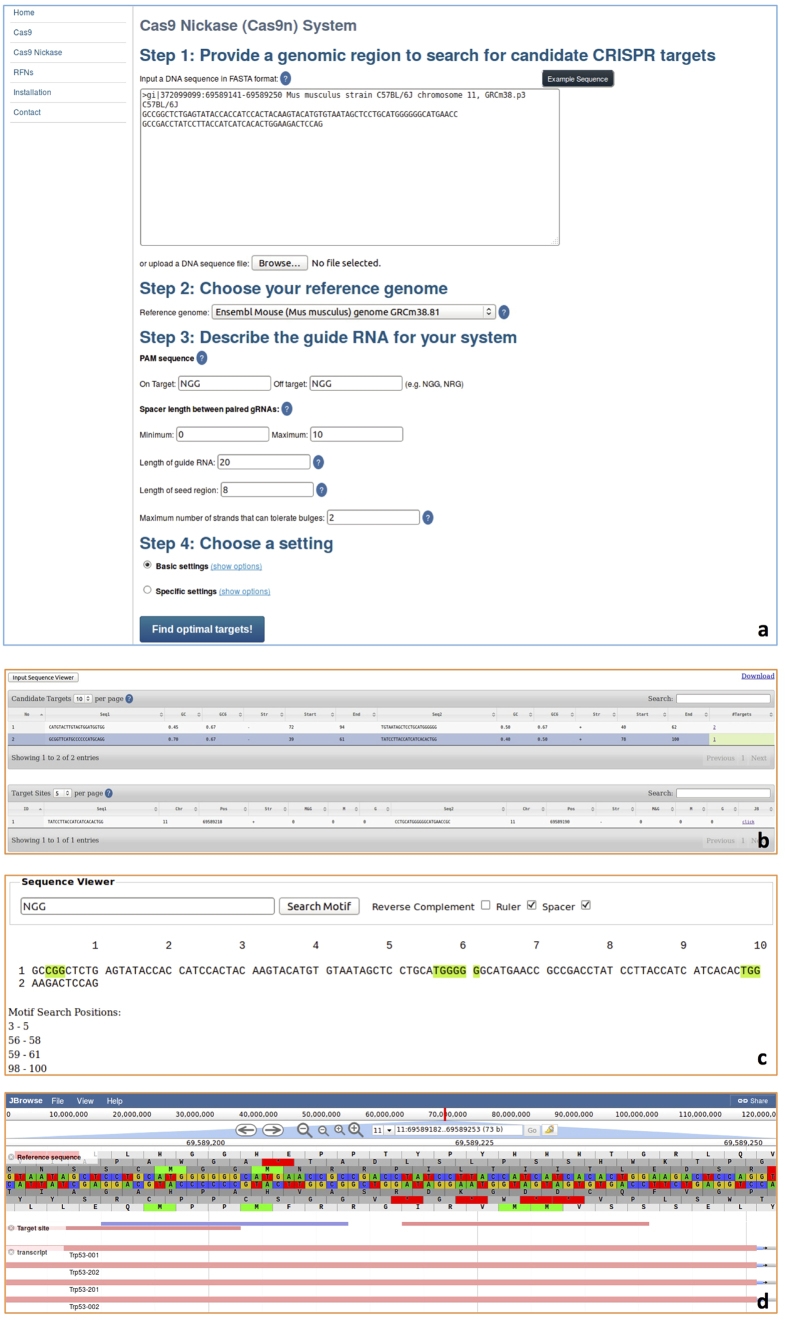
Example settings and result pages for the Cas9n system. Panel (**a)** the settings page for the Cas9n system. Panel (**b)** Table Viewer, which displays a list of target candidates (top) and a list of corresponding target sites in the genome for each target candidate (bottom). Panel (**c)** Sequence Viewer, which shows the user input sequence and highlights the subsequence or pattern specified by a user. Panel (**d)** Genome Browser, which visually displays the target sites and gene annotation.

**Figure 3:**
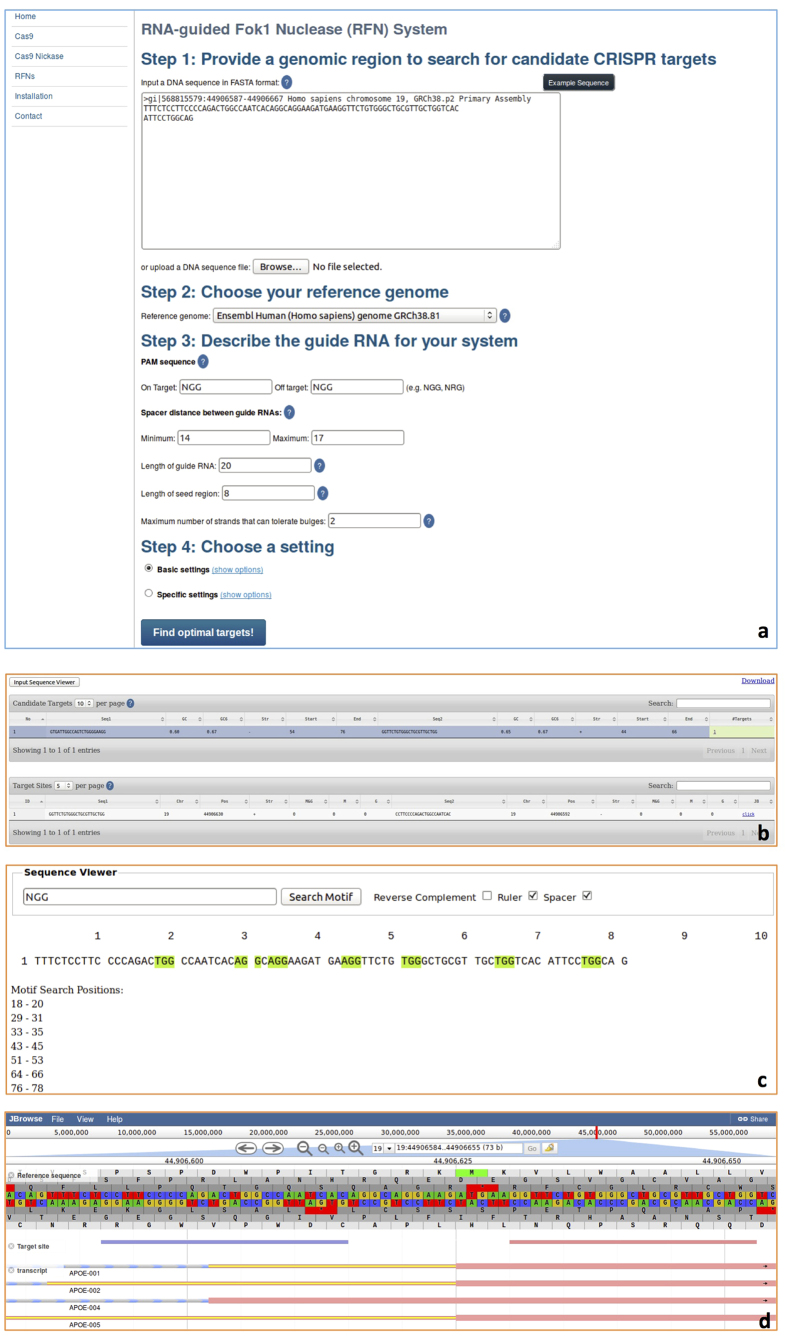
Example settings and result pages for the RFNs system. Panel (**a)** the settings page for the RFNs system. Panel (**b)** Table Viewer, which displays a list of target candidates (top) and a list of corresponding target sites in the genome for each target candidate (bottom). Panel (**c)** Sequence Viewer, which shows the user input sequence and highlights the subsequence or pattern specified by a user. Panel (**d)** Genome Browser, which visually displays the target sites and gene annotation.

Supplementary Fig. S1 shows the *CT-Finder* home page, which has a menu on the left side to support three primary working modes: Cas9, Cas9n, and RFNs systems. If users click “Cas9”, the setting page for the Cas9 system will be displayed (Fig. 1a). Users follow the steps to enter their sequence, select a reference genome, and input their specifications for the target search. For the Cas9 system, users can input the on-target and off-target PAM sequences as well as the length of gRNA and seed region. For indels (gaps) and mismatches, users can choose between “Basic settings” and “Specific settings”. “Basic settings” allows for the maximum number of mismatches and gaps tolerated by off targets to be set. Additionally, “Basic settings” allows the maximum number of mismatches and gaps in the seed region tolerated by off targets to be specified. “Specific settings” includes seed region settings and non-seed region settings. In seed region settings, users can set the maximum number of mismatches, insertions, and deletions respectively tolerated by off targets. In non-seed region settings, users can also set the maximum number of mismatches, insertions, and deletions respectively tolerated by off targets. If users select the “Cas9n” (Fig. 2a) or “RFNs” (Fig. 3a) mode, two additional paired-gRNA settings are presented: one for minimum and maximum spacer length between paired gRNAs and another for the maximum number of strands that can tolerate bulges. The setting pages for RFNs and Cas9n modes differ only by the default minimum and maximum spacer length setting. The setting range of spacer distance between gRNAs in the RFNs system is most effective within a range of 14 to 17 *nt*, while the Cas9n system permits a much wider range, from approximately 0 to 1000 *nt*. After setting all parameters, users may click “Find optimal targets!” to run the program and view the result page.

The result pages for all three systems include a table viewer (*e.g.*, Fig. 1b), a sequence viewer (*e.g.*, Fig. 1c) and a genome viewer (*e.g.*, Fig. 1d). On the table viewer page, if the user selects “Input Sequence Viewer”, a new window will open; in this new “Sequence Viewer” window, users are able to view their target DNA sequence with or without a ruler, with or without a spacer, and in the forward or reverse complementary strand. The “Sequence Viewer” window also includes a function to search for a user-typed pattern or subsequence (*e.g.*, PAM sequence), which will be highlighted wherever it is found in the original sequence. The sequence viewer also helps position subsequences. The table viewer displays a list of target candidates (single gRNAs for the Cas9 system or pairs of gRNAs for the Cas9n and RFNs systems) and relevant information, including which strand it is found on (*i.e.*, forward or reverse), its start position, its end position, GC-content measures, and predicted number of corresponding matched target sites in the genome. A low number of targets is preferred for high specificity. If the value is 1 it will be highlighted. This indicates that the target candidate is highly specific, having only one target site in the whole genome. Each row is clickable to bring up a list of target sites for the chosen target candidate, including information about the sequence, chromosome, strand, start/end position, and number of mismatches and gaps. A link to JBrowse is also included for each target site. Clicking one of these links leads to a genome browser showing the target site within the genomic context. The reference genome sequence is visualized with feature tracks that contain gene annotation information and the target site. Additional tracks may be added on the server or client side.

## Discussion

In applying the CRISPR systems to edit genomes, one of the most important considerations is to improve target specificity. According to recent research, there are two methods to improve target specificity. One method is to search all possible off-target sites in the genome (depending on the experimental off-target features) and then to choose the optimal target candidate that introduces the least abrasive off-target effects^20^. The second method is to use new variants of CRISPR technology to improve target specificity^4^,^7^,^21^. *CT-Finder* combines these two methods to enhance target specificity.

Like its predecessors E-CRISP^9^, GT-Scan^10^, and CRISPRdirect^11^, *CT-Finder* supports a number of experimentally applicable input parameters, such as the PAM sequence, length of seed region, and maximum number of mismatches. However, CRISPR DESIGN^8^ does not allow users to customize these parameters. In addition to the fundamental settings, *CT-Finder* also supports settings for maximum number of gaps. Lin^16^ reported that off-target sites can also tolerate insertions and deletions in addition to mismatches, indicating that gaps need to be considered for off-target analysis. CRISPRdirect also supports gaps, but its users cannot set the length of the seed region and cannot alter the number of mismatches and gaps in the seed region – these features are unique to *CT-Finder. CT-Finder* allows specific settings applicable to the seed region and non-seed region, including the number of mismatches, insertions and deletions, making *CT-Finder* highly flexible to suit future experimental off-target features. *CT-Finder* also, for the first time, introduces the use of JBrowse to integrate and visualize on-target and off-target sites within the genomic context. This visualization interface presents a smoother experience compared to that of other tools, especially in the sense of data visualization and validation. Additional feature tracks, such as gene references, can be conveniently added on the server or client side to satisfy users’ requirements.

Applying new technologies to improve target specificity, *CT-Finder* introduces Cas9n^4^ and RFNs^7^,^21^. Both of these CRISPR variants are based on paired gRNAs and greatly improve target specificity relative to the original single gRNA CRISPR system. CRISPR DESIGN and E-CRISP also support Cas9n or paired gRNAs, but do not allow gaps and the number of strands that can tolerate bulges to be set; *CT-Finder* supports all these settings, leading to more comprehensive and precise off-target searching than other tools. In addition, *CT-Finder* is the first available tool to help users design gRNAs according to any of 3 modes: Cas9, Cas9n and RFNs.

On the other hand, no multivariable scoring algorithm is incorporated currently into *CT-Finder* because there is not enough experimental evidence available to give rational weighting to various factors such as gaps, affecting the likelihood of off-target effects. Prediction of off-target effects is still a new concept and additional research will be necessary to produce more accurate and quantitative algorithms. Furthermore, it is worthy to note that computational algorithms can only predict off-target effects; experimental determination of off-target effects will always be necessary to confirm these predictions.

*CT-Finder* is designed to favor predicted specificity above all other aspects when choosing optimal target candidates. Experimental goals should always be considered when utilizing computational tools, especially ones biased toward specific purposes. In some cases, higher efficiency of cleavage activity may be preferred at the cost of higher risk of off-target effects. In other cases, specificity is crucial, no matter the cost to efficiency.

Beyond prediction of off-target effects, experimental conditions can also play a role in influencing off-target cleavage activity. For example, it has been reported that titrating the amount of SpCas9 delivered can aid in minimizing off-target cleavage activity, but with the drawback of reducing on-target cleavage as well^8^. In addition, cellular environments or genomic factors may, in some cases, despite use of optimal design, preclude efficient targeting of certain genes of interest by CRISPR systems^22^. Other technologies, such as RNAi, may provide a preferable alternative method in these situations.

### Implementation

*CT-Finder* is essentially composed of web interfaces coded in PHP and JavaScript and a backend pipeline implemented in Perl. The web interfaces accept users’ inputs, including many parameter settings, which are then passed to the backend pipeline for data processing and data analysis. The pipeline will generate multiple result files residing in the web server. Afterwards, the results will be displayed through highly interactive web interfaces.

As shown in Supplementary Fig. S2, the backend pipeline contains multiple steps. *CT-Finder* first processes the user-input DNA sequence into a list of subsequences ending with the on-target PAM sequence specified by a user, leading to the creation of a file of all possible target candidates for the given sequence. For the Cas9 option (Supplementary Fig. S2a), these target candidates are single sequences; for either the Cas9n or RFNs option (Supplementary Fig. S2b), the target candidates are paired sequences. Bowtie2^23^, a fast and sensitive read aligner, is then called to align each sequence, consisting of the gRNA target candidate sequence and off-target PAM sequence specified by users, to the reference genome, generating a SAM file. Samtools^24^ is applied to convert the Bowtie2 off-target SAM output into a BAM file, which will be used to separate alignments into either forward or reverse strand alignment files. If the Cas9n or RFNs option is selected, the number of insertions, deletions and mismatches are counted for all individual alignments. Then, the alignments are further separated by chromosomes. The alignments in each chromosome are filtered according to the user-defined minimum and maximum length of the spacer between paired gRNA targets.

For all three options (Cas9, Cas9n, and RFNs), potential off-target sites in alignment files are filtered to exclude alignments that have indels in the PAM sequence. The user may choose either “Basic settings” or “Specific settings” for further filtering of the potential off-target sites. “Basic settings” takes into account the total number of mismatches and gaps of the gRNA sequence and the total number of mismatches and gaps in the seed region. “Specific settings” details restrictions on the number of mismatches, the number of insertions, and the number of deletions for both the seed and non-seed regions. These two settings are applied to narrow the set of potential off-target sites, eliminating any alignments with a greater number of mismatches, insertions, or deletions than the given thresholds. This produces a final set of predicted off-target sites.

## Conclusions

*CT-Finder* is a web service to help users design the optimal gRNAs for Cas9, Cas9n and RFNs systems with a minimal number of off-target effects. The service covers major model organisms, including human, mouse, and Arabidopsis, and can be easily extended to other species. With its capability to accommodate three CRISPR systems, emphasis on target specificity, flexibility in user inputs of multiple parameter settings, and seamless integration into a genome browser, *CT-Finder* will empower many researchers in the gRNA design and optimal target determination of CRISPR-based genome editing.

## Additional Information

**How to cite this article**: Zhu, H. *et al. CT-Finder*: A Web Service for CRISPR Optimal Target Prediction and Visualization. *Sci. Rep.* **6**, 25516; doi: 10.1038/srep25516 (2016).

## Supplementary Material

Supplementary Information
