# Supplementary material for: CT-Finder: A Web Service for CRISPR Optimal Target Prediction and Visualization
Source: Sci Rep. 2016 May 23;6:25516. doi: 10.1038/srep25516 (PMC4876460; doi:10.1038/srep25516)
Supplement: Supplementary Information [file srep25516-s1.pdf]

Supplementary Information For:

CT-Finder: A Web Service for CRISPR Optimal Target Prediction and Visualization

Houxiang Zhu[1], Lauren Misel[1], Mitchell Graham[1] and Chun Liang[1, 2, *]

[1] Department of Biology, Oxford, Ohio, 45056, USA;

[2] Department of Software Engineering, Miami University, Oxford, Ohio 45056, USA;

* To whom correspondence should be addressed: liangc@miamioh.edu

Installation instruction

To install *CT-Finder* locally, users can follow the installation manual on our website (http://bioinfolab.miamioh.edu/ct-finder/interface/Installation.php), which includes the links to download *CT-Finder* virtual machine (http://bioinfolab.miamioh.edu/ct-finder/vmware/Ubuntu.tar.gz) and *CT-Finder* source code (https://sourceforge.net/projects/ct-finder/files/latest/download).

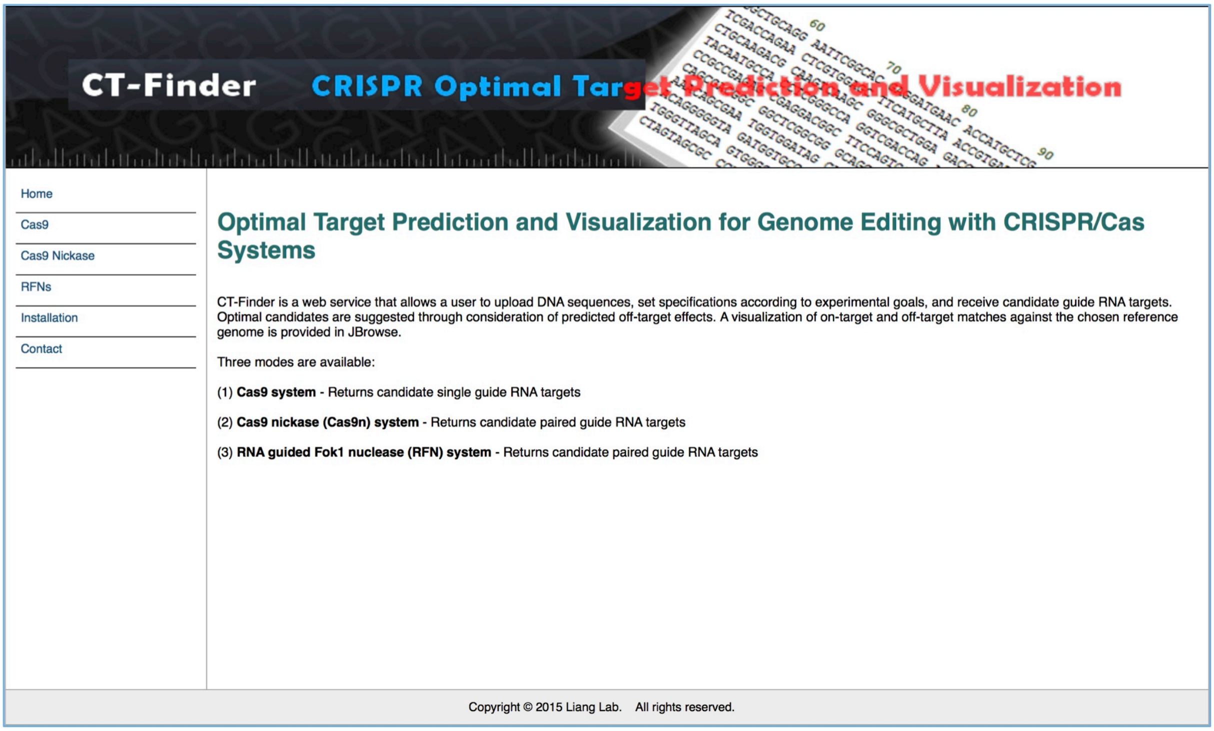

Supplementary Figure S1. The home page of *CT-Finder* web service, including three primary working modes: Cas9, Cas9n, and RFNs systems.

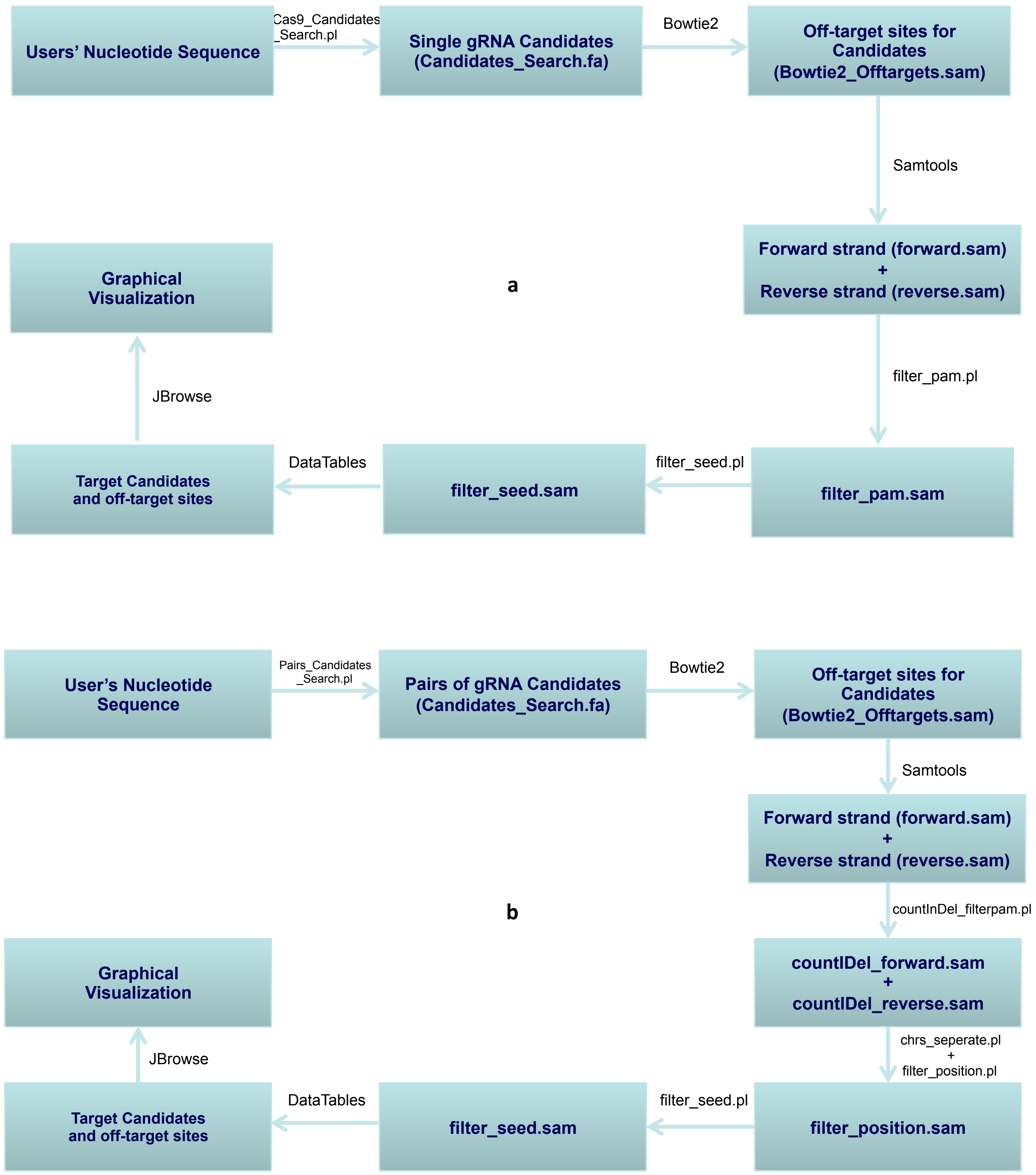

Supplementary Figure S2. Workflow of *CT-Finder* web service that incudes web interfaces and a backend bioinformatics pipeline. Panel a: the workflow for finding the optimal target candidates for Cas9. Panel b: the workflow for finding the optimal target candidates for Cas9n and RFN.
